# Multicolor Combinatorial Probe Coding for Real-Time PCR

**DOI:** 10.1371/journal.pone.0016033

**Published:** 2011-01-14

**Authors:** Qiuying Huang, Linlin Zheng, Yumei Zhu, Jiafeng Zhang, Huixin Wen, Jianwei Huang, Jianjun Niu, Xilin Zhao, Qingge Li

**Affiliations:** 1 Engineering Research Centre of Molecular Diagnostics of the Ministry of Education, Department of Biomedical Sciences and the Key Laboratory of Cell Biology and Tumor Cell Engineering of the Ministry of Education, School of Life Sciences, Xiamen University, Xiamen, China; 2 Department of Microbiology, Xiamen Centre for Disease Control and Prevention, Xiamen, China; 3 Public Health Research Institute, New Jersey Medical School, University of Medicine and Dentistry of New Jersey, Newark, New Jersey, United States of America; St. Petersburg Pasteur Institute, Russian Federation

## Abstract

The target volume of multiplex real-time PCR assays is limited by the number of fluorescent dyes available and the number of fluorescence acquisition channels present in the PCR instrument. We hereby explored a probe labeling strategy that significantly increased the target volume of real-time PCR detection in one reaction. The labeling paradigm, termed “Multicolor Combinatorial Probe Coding” (MCPC), uses a limited number (n) of differently colored fluorophores in various combinations to label each probe, enabling one of 2*^n^*-1 genetic targets to be detected in one reaction. The proof-of-principle of MCPC was validated by identification of one of each possible 15 human papillomavirus types, which is the maximum target number theoretically detectable by MCPC with a 4-color channel instrument, in one reaction. MCPC was then improved from a one-primer-pair setting to a multiple-primer-pair format through Homo-Tag Assisted Non-Dimer (HAND) system to allow multiple primer pairs to be included in one reaction. This improvement was demonstrated via identification of one of the possible 10 foodborne pathogen candidates with 10 pairs of primers included in one reaction, which had limit of detection equivalent to the uniplex PCR. MCPC was further explored in detecting combined genotypes of five β-globin gene mutations where multiple targets were co-amplified. MCPC strategy could expand the scope of real-time PCR assays in applications which are unachievable by current labeling strategy.

## Introduction

High-throughput, yet simple, reliable, and cost-effective molecular testing methods are crucial for efficiently utilizing vast quantities of genetic information to elucidate mechanisms of disease, identify potential targets for therapy and diagnosis, track the identity or origin of subject organisms, and personalize treatment protocols for individual patients. Among the many valuable tools that have been explored in nucleic acid-based testing, real-time PCR-based methods have unique advantages because they allow real-time, kinetic detection of amplified product accumulation through fluorescence intensity change in a closed-tube setting, eliminating the need for post-amplification manipulation, and thereby markedly reducing the chances of cross-contamination [1]. The high reproducibility, quantification ability and ease of use have rendered real-time PCR suitable in a broad range of applications [2]. However, the availability of limited number of fluorescence labels that can be monitored simultaneously has hampered the use of multiple probes for multiplex assays. Thus, the target volume of real-time PCR-based methods is usually low irrespective of the probe type used. Real-time PCR assays for multiple targets detection have been reported [3], [4], [5], but few, if any, of these reported assays could detect more than four or five targets in a single reaction. A modified form of fluorescent PCR approach, which detects the end products by fluorometric scanning after amplification, instead of by real-time, dynamic monitoring during the amplification process, improved the number of targets detectable in a single tube to six [6]. Such improvement, however, is still far less robust than is needed for high-throughput detection. Therefore, despite the unique advantages of real-time PCR-based nucleic acid detection [1], [2], [7], the traditional one-color, one-probe labeling strategy creates a low target volume bottleneck that greatly limits its application to multiplex detection and genotyping.

In an effort to overcome the above shortfall of real-time PCR, we have designed a labeling strategy termed multicolor combinatorial probe coding (MCPC) that enables the labeling of a large number of different probes with a limited number of differently colored fluorophores. Theoretically, this simple, multicolor combinatorial labeling format, which resembles the same logic used in the so-called chromosome painting technique [8], enables the inclusion of as many as 2*^n^*-1 different probes in a single assay, utilizing only *n* differently colored fluorophores. By comparison, classical one-color, one-probe approaches are limited to the use of *n* probes, each labeled with one of the *n* differently colored fluorophores.

A key feature of MCPC strategy is that it targets for situations where only one out of many potential targets need to be detected. Such situations are commonly encountered in detection of infective agent at the normally sterile sites (e.g. blood), molecular tying/identification for a specific causative pathogen from a large list of possible candidates after the microorganism has been isolated (e.g. purified to a single colony or plaque) and genetic disease diagnosis/screening where co-existence of multiple genotypes in the same sample under these situations is very rare, if not impossible. Under such circumstances, MCPC allows identification of any one potential target from multiple possible candidates (e.g. 1 of 15 or 1 in 31) in a single reaction. In a previous study, we established a method to differentiate 8 different foodborne pathogens using 4 different fluorophores using the MCPC strategy [9]. Although the target number of that study is larger than the traditional one-color, one-probe labeling strategy, it does not reach theoretically detectable number of MCPC. Moreover, the analytical sensitivity of MCPC decreased significantly when compared with the corresponding uniplex PCR.

In the present work, we further explore the MCPC concept in different applications and validate it with real samples collected from a variety of sources. We first demonstrated that the theoretical upper limit, e.g. 15 combinations, could be achieved experimentally with a 4-color real-time PCR platform. We then showed that MCPC could be performed in a multiple-primer-pair format through a novel primer dimer alleviating strategy. Such a strategy allowed detection of each of the 10 possible targets in one reaction with limit of detection equivalent to uniplex PCRs. Moreover, we expanded the applicability of MCPC to situations when more than one target is co-amplified and needs to be differentiated in one reaction.

## Results

### Design of MCPC-labeled Probes

In classical real-time PCR, each differently colored fluorophore is used to label one probe that detects one genetic target. In such a setting, four differently colored fluorophores are restricted to label four probes that detect or distinguish only four targets. Consequently, both the number of distinguishable fluorophores available for probe labeling and the number of fluorescence detection channels that are present in a real-time PCR instrument for signal acquisition pose a target number bottleneck for multiplex detection.

**Figure 1:**
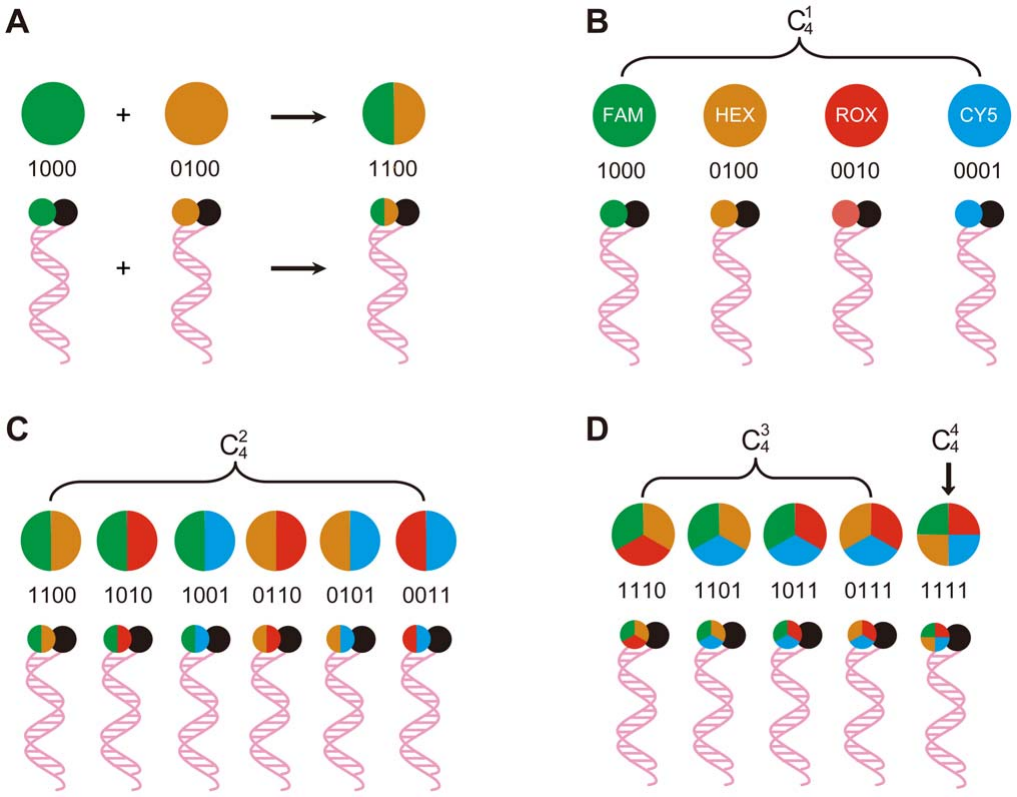
Schematic representation of the Multicolor Combinatorial Probe Coding (MCPC) strategy for multiplex, real-time PCR genotyping using displacing probes. Four differently colored fluorophores (FAM: green; HEX: orange; ROX: red; and Cy5: blue) and one universal quencher (DABCYL: black) are used to depict the principal of MCPC. **A**) Illustration of how half the molecules of a third probe in an assay can be labeled in one color and the other half of the molecules of the third probe can be labeled with a second color, producing a single probe that generates a two-color signal when it hybridizes to its target. **B**) Four probes, each labeled with a different single color. **C**) Six probes, each labeled with a unique combination of two of the four colors. **D**) Four probes, each labeled with a unique combination of three of the four colors, and one probe labeled with all four colors. For each fluorophore, a unique four-digit code, MCPC signature, is given, i.e., FAM is 1000, HEX is 0100, ROX is 0010, Cy5 is 0001. For the combined fluorophores, the MCPC signatures are also combined for coding.

To enable a large number of probes to be labeled, and many targets to be distinguished with a limited number of fluorophores and fluorescence detection channels, we used differently colored fluorophores in various combinations to label individual probes. For example, two differently colored fluorophores can be used to distinguishably label three different probes, i.e., two probes can each be labeled with one color, a third probe can be labeled with both colors. If four different fluorophores can be used, four probes can each be labeled with a unique (single) color, six probes can each be labeled with a unique combination of two of the four colors, four probes can each be labeled with a unique combination of three of the four colors, and one probe can be labeled with all four colors, resulting in a total of 15 uniquely labeled probes (Figure 1).

To make one probe to be labeled with a certain combination of different colors, a straight but technically challenging way is to chemically conjugate a single probe with a mixture of different fluorophores. An alternative but more practical way is to mix equal molar quantities of probes each labeled with a single but different colors to function as a single probe set labeled with different colors. According to the combination rule, if each fluorophore is given a digital code, then each of the 15 combinatorially labeled probes will have a unique digital code (MCPC signature), e.g., 1000 for FAM, 0100 for HEX, and 1100 for FAM+HEX combination. The MCPC signature can help identify each probe and its corresponding target. In general, the maximum number of probes that can be uniquely labeled with *n* differently colored fluorophores is 2*^n^*-1.

### Attainability of Maximal Coding Limit of MCPC

**Figure 2:**
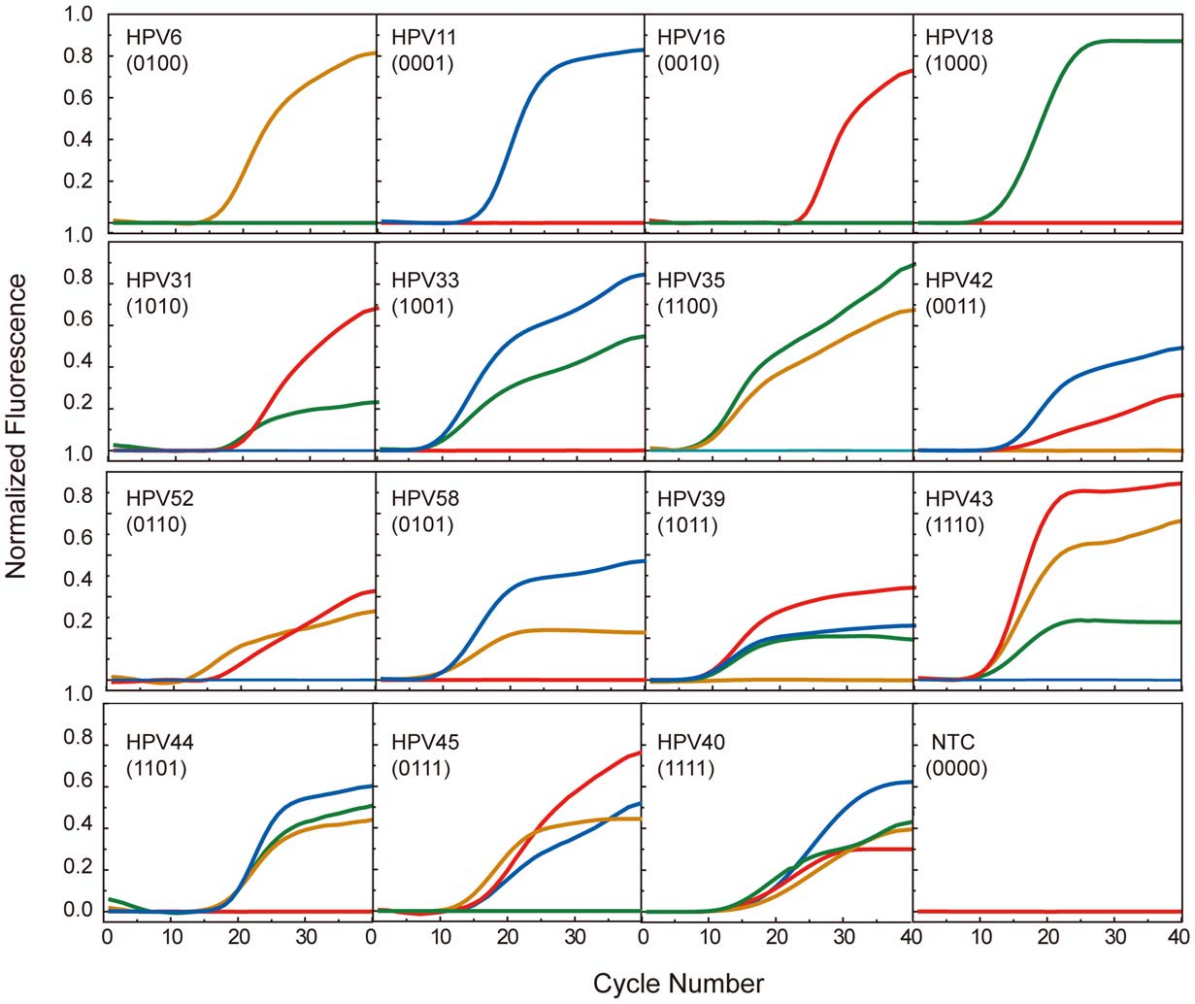
Real-time PCR typing of HPV with MCPC. One pair of primers and 15 probe sets were mixed in each reaction tube for identification of one of the 15 possible viral types in a real-time PCR setting, as described in the Material and Methods section. Fluorescence signals were recorded in all four channels of the fluorometric thermocycler (FAM: green; HEX: orange; ROX: red; and Cy5: blue). The MCPC signature is given for each type.

We first tested whether we could achieve the theoretical maximal coding limit of MCPC. To do so, we mixed 1 primer pair and 15 probe sets in a single PCR tube to see whether we can simultaneously detect the existence of each of the 15 possible HPV types. According to the MCPC strategy, each type of HPV would be detected with a unique probe set and would thus display a unique amplification profile accordingly. As expected, the amplification results showed the exact concordance between the HPV type and the MCPC amplification profile generated from the corresponding probe set (Figure 2) despite varied C_q_ (quantification cycle) values among the 15 types of HPV due to differences in starting template concentrations for each type. We further tested whether accurate typing of HPV could be realized in different template concentrations by varying template copy numbers. Repeatable and reliable typing was achieved when plasmid template copy numbers varied from 10^2^ to 10^9^ copies per reaction for each of the 15 HPV types. Taken together, the HPV typing results demonstrate that the theoretical upper limit of MCPC can be realized experimentally.

### Improved Analytical Sensitivity in MCPC

In contrast to the HPV typing where only a single primer pair was used to amplify a single target, multiple primer pairs are often used to amplify multiple targets in multiplex assays. To test the applicability of MCPC in such a setting, a model detection system was studied where 10 primer pairs were used to amplify 10 species-specific genes for identification one of the 10 potential foodborne pathogens in one reaction. As previous observed in an 8-plex real-time PCR, the analytical sensitivity was 100–1000 folds lower than the corresponding uniplex PCRs [9]. One possibility for such low sensitivity may derive from formation of primer dimer in such a heavily multiplexed setting causing significant decrease of the amplification efficiency, thereby resulting in substantial loss in the overall analytical sensitivity. To overcome this problem, we introduced the Homo-Tag Assisted Non-Dimer (HAND) system to alleviate primer dimer formation in the multiple-primer-pair format of MCPC (in contrast to one-primer-pair format in the case of HPV typing). The HAND system was designed in such a way that if a primer dimer forms, it will exist in the form of two separate hairpin structures instead of one double-stranded structure. Once formed, the hairpin structure hinders the primer annealing for further amplification and therefore primer dimer, if formed, could only be produced in a linear way [10].

**Figure 3:**
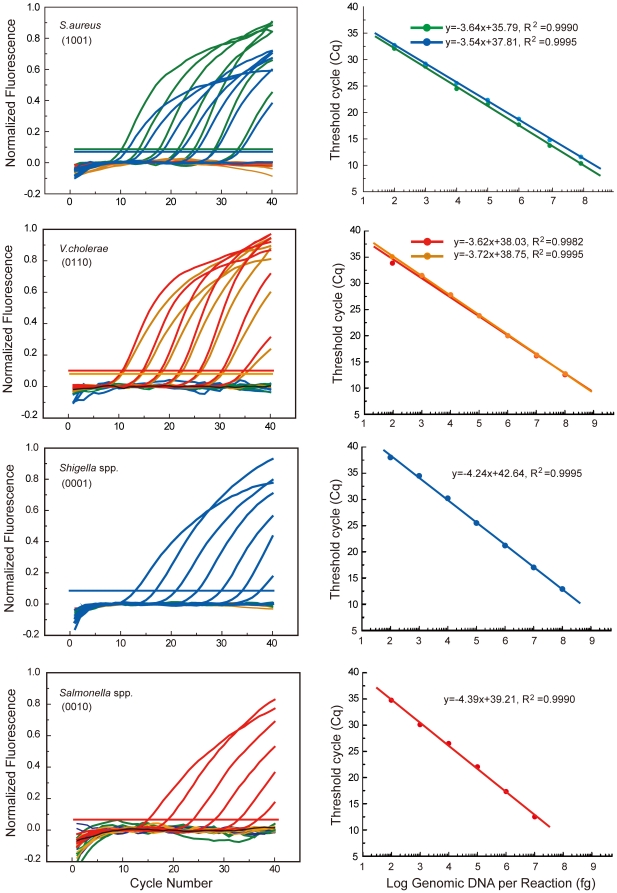
Representative results of real-time PCR quantification with MCPC for *S*. *aureus*, *V. cholerae*, *Shigella* spp. and *S. typhi*. Purified DNA templates were 10-fold serially diluted from 100 ng to 100 fg and water was used as non-template control. The MCPC signature is given for each bacterium type. FAM: green; HEX: orange; ROX: red; and Cy5: blue.

Our results showed that adoption of HAND improved analytical sensitivity of the 10-plex MCPC assay to the same level of the uniplex real-time PCR (Table 1 and Figure 3), which was 100- to 1000-fold higher than the previous 8-plex PCR assay [9]. The specificity of this 10-plex MCPC system was validated using a blind test of 157 bacterial samples including the 10 foodborne pathogens and 14 other types of pathogens (Supplementary Table S1). The results showed that all 10 foodborne pathogens were correctly identified and no false-positive signal was observed when samples contained 14 non-related pathogens. The usefulness of this 10-plex MCPC assay was recognized by assembling the reagents into a test kit for routine testing in Xiamen CDC for screening of food-poisoning bacteria. Taken together, our results demonstrated that while multiplex MCPC assay was as specific as a classical probe-based real-time PCR assay; it could also be as sensitive as a uniplex PCR assay when combined with HAND system for primer design. This improvement ensured the robustness of MCPC in clinical settings.

**Table 1:** Quantitative detection of 10 bacterial strains by MCPC in a single tube.

Bacterium strains	Coding format	Limit of detection (CFU/reaction)	Dynamic range	R^2^
*B. Cereus*	FAM	5	100 fg∼100 ng	0.9999
*E. Coli* O157:H7	HEX	5	100 fg∼100 ng	0.9989
*S.* typhi.	ROX	5	100 fg∼10 ng	0.9990
*Shigella* spp.	Cy5	5	100 fg∼100 ng	0.9995
*V*. *parahaemolyticus*	FAM + HEX	10	100 fg∼100 ng	0.9995/0.9994
*S. pyogenes*	FAM + ROX	10	100 fg∼100 ng	0.9995/0.9992
*S*. *aureus*	FAM + Cy5	10	100 fg∼100 ng	0.9990/0.9995
*V. cholerae*	HEX + ROX	10	100 fg∼100 ng	0.9982/0.9995
*Y. enterocolitica*	HEX + Cy5	10	100 fg∼100 ng	0.9996/0.9995
*L*. *monocytogenes*	ROX + Cy5	10	100 fg∼100 ng	0.9995/0.9989

### Simultaneous Detection of Multiple Mutations Using MCPC Platform

While MCPC strategy can be readily implemented when one of many potential targets need to be amplified and detected, it can be challenging when two or more targets are co-amplified and need to be differentiated in one reaction. In the latter case, color overlaps can occur between singly labeled, doubly labeled, and multiply labeled probes, and thus the MCPC profiles can be too complex to discriminate.

To explore whether MCPC can also be used is some cases where more than one target need to be simultaneously detected, a model study was performed by genotyping five common Chinese β-thalassemia mutations in β-globin gene [11]. These mutations are distributed in a 1.6-kb region, and both homozygous and double heterozygous mutations (compound mutation) can cause β-thalassemia, one of the most serious genetic disorders worldwide [12]. In this assay, three regions were co-amplified to cover the five mutations and a conserved region in the β-globin gene and six MCPC probe sets were used to target the respective mutations as well as the conserved region. Possible combined genotypes to be determined included the wild-type, five heterozygous mutations, 10 double heterozygous mutations and 5 homozygous mutations. Each of these genotypes could be assigned with an MCPC signature (Table 2).

**Table 2:** Twenty-one possible genotypes and their MCPC signatures.

Signature number	Genotype	MCPC signature^a^					
		FAM	HEX		ROX		Cy5
wild-type and heterozygous carrier							
1	Wild-type	0		0	0		2
2	c.316–197C>T	1		0	0		2
3	c.52A>T	0		1	0		2
4	c.-78A>G	0		0	1		2
5 (9)^b^	c.216_217insA	1		0	1		2
6 (13)^b^	c.125_128delTCTT	0		1	1		2
homozygous and compound heterozygous mutations							
7	c.[316-197C>T]+[316-197C>T]	2		0	0	2	
8	c.[316-197C>T]+[52A>T]	1		1	0	2	
9 (5)^b^	c.[316-197C>T]+[-78A>G]	1		0	1	2	
10	c.[316-197C>T]+[216_217insA]	2		0	1	2	
11 (14)^b^	c.[316-197C>T]+[125_128delTCTT]	1		1	1	2	
12	c.[52A>T]+[52A>T]	0		2	0	2	
13 (6)^b^	c.[52A>T]+[-78A>G]	0		1	1	2	
14 (11)^b^	c.[52A>T]+[216_217insA]	1		1	1	2	
15	c.[52A>T]+[125_128delTCTT]	0		2	1	2	
16	c.[-78A>G]+[-78A>G]	0		0	2	2	
17	c.[-78A>G]+ [216_217insA]	1		0	2	2	
18	c.[-78A>G]+[125_128delTCTT]	0		1	2	2	
19	c.[216_217insA]+ [216_217insA]	2		0	2	2	
20	c. [216_217insA]+[125_128delTCTT]	1		1	2	2	
21	c.[125_128delTCTT]+ [125_128delTCTT]	0		2	2	2	

a In MCPC signature, “0” represents no signal, “1” represents signal from one haploid, “2” represents signal either from two haploids or from one haploid detected by two different probes but labeled with the same fluorophore.

b The signature number in the parenthesis denotes the degenerate signatures that are not distinguishable between the two possible genotypes without parental genotype information.

**Figure 4:**
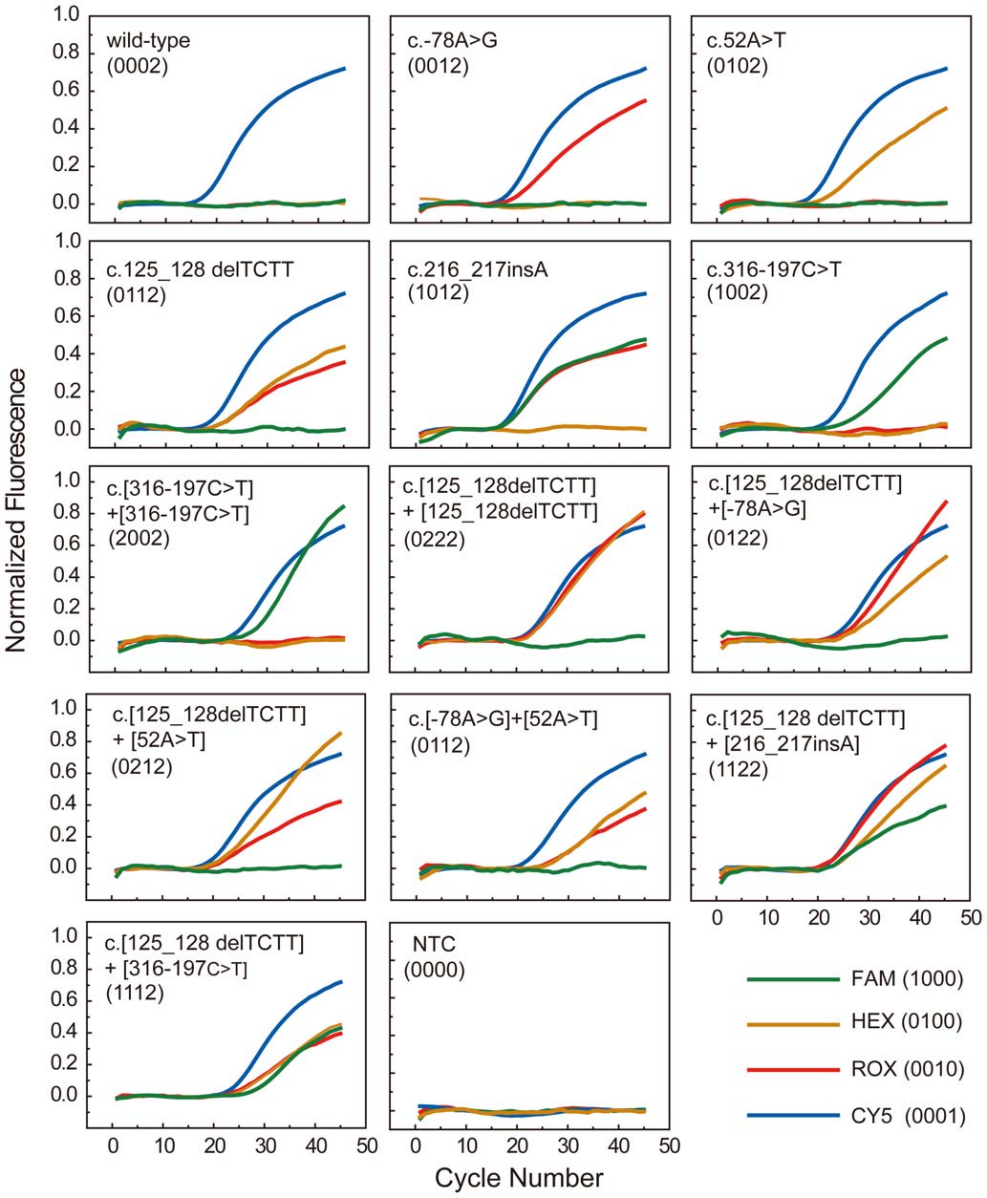
Real-time PCR genotyping of 13 different β-globin genotypes in clinical samples with MCPC. The assigned genotypes are listed with MCPC signatures and genotypes.

The MCPC signatures listed reflect that the signal intensity at the end of cycle plays a key role in discrimination of genotypes that can be reported by the same color combination. For example, both heterozygous and homozygous mutation of c.316-197C>T have FAM and Cy5 signals, but the heterozygous mutation has a MCPC signature of 1002 whereas the homozygous mutation has an MCPC signature of 2002. This difference was shown by the real-time PCR profiles where lower FAM signal at the end of cycle than Cy5 was seen in the heterozygous mutation whereas higher FAM signal was seen in the homozygous mutation (Figure 4). Such a difference is due to the existence of the two copies of mutant allele in the homozygous sample contributing significantly to FAM fluorescence intensity, thereby altering the relative fluorescence intensity ratio of FAM to Cy5 intensity observed in heterozygous samples which has only one copy of mutant allele. Similarly, the MCPC signature of c.125_128delTCTT heterozygous mutation is 0112 while the homozygous mutation is 0222, therefore the heterozygous mutation has lower HEX and ROX signal than the homozygous mutation at the end of cycle. Similar results can also be seen in compound mutations. For example, compound mutation of c.[316-197C>T]+[125_128delTCTT] has a MCPC signature of 1112, while compound mutation of c.[216_217insA]+[125_128delTCTT] has a MCPC signature of 1122. The former therefore showed a lower ROX signal than the latter in real-time PCR profiles. Thus, the MCPC strategy can work even when more than one target is amplified when the signal intensity ratios between colors are used for differentiation.

According to Table 2, degenerate signatures occur between 3 pairs of genotypes, i.e., No 5 versus No. 9, No. 6 versus No. 13, and No. 11 versus No. 14. These genotypes could not be discriminated by their real-time PCR profiles because both color types and color intensities would be completely overlapped between the two genotypes in each of the 3 pairs. However, these degenerate signatures pose no influence in clinical settings. In clinical practice, prenatal diagnosis is conducted only when both parents are heterozygous with known genotypes [13], therefore it is straightforward to know the exact genotype of the natal by simply checking one of the parental genotypes. Since none of the degenerated signature pairs share a common genotype, parental carrier genotype will help unequivocally distinguish the degenerated signature pairs. If the assay is used for population-based symptomless carrier screening, the degenerate genotypes will again cause no confusion simply because these degenerate signatures involve discrimination between carrier and disease (homozygous or compound heterozygous alleles cause disease) states whereas the disease state is already excluded from the screening.

Finally, the established MCPC assay was blindly tested with 239 clinical samples that have been previously analyzed by the reverse dot-blot method, 13 different genotypes (Figure 4) representing 12 patients, 98 carriers, and 129 normal samples (Table S2) were accurately identified. The limit of detection of the triplex MCPC assay obtained from six genotypes (one wild-type and five heterozygotes) was 10 pg of genomic DNA (equivalent to approximately 3 copies of human diploid) per reaction, which was equivalent to the ultimate limit of detection of any uniplex PCR. Thus, when signal intensity is considered and optimized, MCPC can be used in assays when more than one of the multiple candidate alleles is co-amplified and co-detected.

## Discussion

By using a limited number of fluorophores in various combinations to label the probes, rather than labeling each probe with a different fluorophore, MCPC strategy allows the detection in a single reaction one of a maximal number of 2*^n^*-1 targets where *n* is the number of differently colored fluorophores used or the number of fluorescence acquisition channels present in the PCR instrument. Such a labeling strategy confers a significant increase in multiplex real-time PCR throughput and allows identification of each one possible target from 15, 31, or 63 different candidate genotypes in a single reaction tube, utilizing a 4-, 5-, or 6-color instrument, respectively. Thus, the number of identifiable targets in real-time PCR reactions can be increased beyond both the number of individual fluorescent colors currently available for probe labeling and the number of fluorescent channels an instrument has for signal acquisition.

According to MCPC principle, the fluorescence intensities of different fluorophores used to label one probe are presumed to be basically equal. If however, the fluorescence intensities of different fluorophores are not kept constant, increased coding signatures can be constructed. In this regard, two advanced coding strategies, permutational coding and exponential coding, can be envisioned. In the permutational coding format, both the type of color combination and the relative fluorescence intensity of each color within a combination are used as coding elements. For example, two colors, A and B, can only encode the A, B, and AB coding patterns by a classical combinatorial labeling strategy. A permutational format, however, would enable four coding patterns to be used: A, B, AB (where A>B), and BA (where A<B). When ***n*** colors are used and two different levels of intensity are employed, the maximum number of permutational coding patterns is Σ [*P_n_^i^*] (where i = 1 to *n*). That would permit coding of 64 probes using four colors and 325 probes using five colors. In the most advanced exponential coding format, where multiple levels of fluorescence intensity can be distinguished for each color, the maximum number of probes that can be coded by *n* colors, each having ***x*** levels of fluorescence intensity, is expressed by: P*_n_* ^1^ + Σ [x^i^ P*_n_* ^i^] (where i = 2 to *n*). In this format, a 4-color, 10-fluorescent intensity labeling scheme would permit coding of: P_4_^1^ + (10^2^ P_4_^2^ + 10^3^ P_4_^3^ + 10^4^ P_4_^4^) = 265,204 probes. Although the discrimination of so many coding patterns currently appears to be an experimentally formidable task, the new coding strategy should theoretically enable the labeling of unlimited numbers of probes with only a handful of fluorophores. A computer-aided pattern recognition may help distinguish the many fluorescent fingerprints generated by the advanced MCPC assays. According to MCPC, a large number of probes are included in one reaction tube. These probes, however, did not cause unbearable background when dark quencher-labeled probes, such as molecular beacons or displacing probes, were used as demonstrated in this study. Also, the presence of multiple primers did not cause decrease in amplification efficiency by taking advantage of HAND system and therefore high analytical sensitivity and wide dynamic range of detection could be achieved. These features enable MCPC to retain the quantitative nature of real-time PCR while substantially enhance its qualitative ability.

Although we showed that MCPC can be used in situations where more than one targets need to be simultaneously detected when signal intensity ratios are considered (Figure 4 and Table 2), one of the main drawbacks of MCPC strategy still lies in its less or even unsuitability for simultaneous detection of multiple targets in one reaction. Such a limitation makes MCPC best suited for applications such as genetics disease screening where diseases are elicited by only one of many alleles, rapid determination of fusion type in leukemia, pathogen identification of blood-borne or other normally sterile site infections that are often caused by one of many possible pathogens, and identification of pre-purified causative organisms that may belong to a large panel of candidates. One way to partially overcome this drawback is to use only one combination to label all the probes. For example, if combination C_4_^2^ is only used to label the probes, six different probes can be each labeled with two different fluorophores for six targets and any fluorescence overlapping occurred could be discriminated. This is because any possible overlapping could be easily resolved by normalization with the amplification profile from each individual target. However, this modified approach suffers the decrease in the number of targets that can be detected. One better way is to use digital PCR [14], where single, partitioned DNA molecule or cell served as templates for individual multiplex PCR. Such a platform can prevent the existence of mixed samples (species or multiple cells) in one reaction from occurring. With commercially available microfluidic devices to perform digital PCR [15], [16], [17], ten thousands MCPC-based real-time PCR reactions might be performed in parallel. It is thus conceivable that MCPC could be combined with such systems for further improvement in assay throughput. Despite the difficulties in multiple targets detection that may necessitate a second PCR assay, real-time PCR combined with MCPC labeling strategy can still be a valuable pre-screening step to determine the existence of a large spectrum of possible targets, ruling out all negative samples, identifying most genotypes of interest, and narrowing positive outcomes to a few possibilities.

In conclusion, MCPC enables the coding of large numbers of probes that can recognize one target among many potential candidates. By combinatorial use of color type, fluorescence intensity, and C_q_ value in real-time PCR settings, much more fluorescence profile patterns (fingerprints) can be generated and used to distinguish specific genotypes. This labeling strategy significantly improves the target volume of classical real-time PCR in multiple target identification, quantification, and even simultaneous detection when armed with emerging template partitioning devices.

## Materials and Methods

### Probe Preparation

Two types of probe were used. The first type of probe, displacing probe, was used when high specificity was required. Displacing probe can discriminate between the target that is perfectly complementary to the probe and targets that possess a single mismatched nucleotide [18]. They were used in differentiation of HPV types and detection of β-globin gene mutations. The second type of probe, shared-stem molecular beacon, was used when high sensitivity was required. Shared-stem molecular beacons were designed by utilizing one or both of the stem sequences as complementary portion to the targets [19]. These probes were used in differentiation of foodborne pathogens by targeting their respective genes.

All probes were prepared based on the MCPC rule, i.e., multicolor probes were prepared by individually labeling the same probe with different fluorescent colors followed by mixing equal molar quantity of each colored probe to produce a target-specific multicolored probe set. All DNA oligonucleotides were commercially synthesized and purified by Sangon (Shanghai, China). The concentrations of the oligonucleotides were measured by ND-1000 UV-VIS spectrophotometer (NanoDrop Technologies Inc, Wilmington, USA).

### Differentiation of 15 HPV Types through a 4-color MCPC Strategy

Recombinant plasmids containing the HPV L1 gene fragment were used to build an HPV test panel containing the following 15 viral types: 6, 11, 16, 18, 31, 33, 35, 39, 40, 42, 43, 44, 45, 52, and 58. Two 85-basepair DNA fragments (with a 20-basepair overlap) for each viral type containing a portion of the L1 gene were designed on the basis of sequence information derived from the HPV database (http://ncv.unl.edu/HPV/Database.html) and amplified by the overlap extension technique [20]. Each fragment was then cloned into plasmid pMD-18T (Takara, Shanghai, China) to produce PCR templates. GP5+/GP6+ consensus primers were used to amplify a fragment of approximately 150 bp in the L1 open-reading frame [21]. A set of probes labeled with different combinations of four fluorophores were designed to discriminate each HPV type (Table S3).

The 4-color, real-time PCR differentiation of 15 HPV types was setup as below. Real-time PCR was performed in a final reaction volume of 25 µL containing 5 µL plasmids (concentration not calibrated and might vary with different species), 10 mM Tris-HCl (pH 8.6), 50 mM KCl, 2.0 mM MgCl_2_, 200 µM dNTPs, 10 pmol each of the primer pair, 5 pmol each of the 15 probe sets (Table S1), and 1.0 U of *TaKaRa Taq* (Takara, Dalian, China). PCR conditions were as follow: preheating for 3 min at 95°C before 10 cycles of 95°C for 15 s, 48°C for 20 s, and 72°C for 20 s; followed by 40 cycles of 95°C for 15 s, 38°C for 20 s, and 72°C for 20 s. Fluorescence was measured at 38°C from the FAM, HEX, ROX, and Cy5 channels in the Mx3000P fluorometric thermocycler (Stratagene, La Jolla, USA).

### Identification of 10 Foodborne Pathogens with Improved Analytical Sensitivity by HANDS

Reference strains of 10 common foodborne pathogens were obtained from the collections of Shenzhen Center of Disease Control and Prevention (Shenzhen CDC). The 10 species were *Bacillus cereus, Escherichia coli O157:H7, Salmonella typhi, Shigella* spp., *Vibrio parahaemolyticus, Listeria monocytogenes, Staphylococcus aureus, Vibrio cholerae, Streptococcus pyogenes,* and *Yersinia enterocolitica*. All species were grown in nutrient broth except for *S. pyogenes* (grown in dextrose meat infusion broth). After incubation at 37°C (225 rpm shaking) overnight, DNA was extracted from each culture using AxyPrep™ Bacterial Genomic DNA Miniprep Kit (Axygen Biosciences, Union City, CA). The DNA extracted from the culture was quantified by measuring the UV absorbance at 260 nm with the ND-1000 UV-VIS spectrophotometer.

The MCPC assay was developed basing on the uniplex real-time PCR detection kits for the ten foodborne pathogens. These kits were designed to target one specific gene of each causative pathogen with one specific primer pair and one shared-stem molecular beacon probe (Table S4). An important feature of these kits is that they all work under the same thermocycling conditions so that any combinations of them are compatible in multiplex PCR (up to quadruplex). In the present work, the same primer and probe sequences were used except that a universal sequence was added to the 5′-end of the primers according to the principle of HANDS [10]. The tag was also used as a universal primer in the reaction. The sequence of the tag was selected so that it was not homologous to any known bacterial strains as confirmed by BLAST analysis (http://blast.ncbi.nlm.nih.gov/Blast.cgi).

The 4-color, real-time PCR identification of 10 foodborne pathogens was setup as follow. Real-time PCR was performed in a final reaction volume of 25 µL, containing 5 µL of extracted DNA, 10 mM Tris-HCl (pH 8.6), 50 mM KCl, 3.0 mM MgCl_2_, 250 µM dNTPs, 0.01 µM each of 10 tagged primer pairs, 0.2 µM universal primer, 0.2 µM each of 10 probe sets (Table S4), and 1.2 U of *TaKaRa Taq*. PCR conditions were as follow: denaturation at 95°C for 3 min followed by 10 cycles of 95°C for 15 s, 55°C for 20 s, and 72°C for 30 s and another 40 cycles of 95°C for 15 s, 50°C for 20 s, and 72°C for 20 s. Fluorescence was measured at 50°C from the FAM, HEX, ROX, and Cy5 channels on the Mx3000P fluorometric thermocycler.

A blind test of 157 bacterial samples including 10 foodborne pathogens and 14 other pathogens was performed to validate the MCPC assay. Twenty of the 157 strains were isolated from food products by Shenzhen CDC; the rest were all clinical isolates. All bacteria were previously identified by colony morphology, biochemical properties, and immune agglutination. Template DNA was prepared from each culture using the kit as described above.

### Detection of Multiple β-globin Gene Mutations

To detect five β-thalassemia mutations in β-globin gene, three primer pairs were designed to co-amplify three regions containing these mutations. All primers were added with a universal tag sequence at their 5′ ends according to HANDS as previously described. Six probes were labeled with fluorophores according to MCPC (Table S5).

The 25-µL reaction contained 5 µL of DNA template (50 ng genomic DNA or 5.0×10^6^ copies of plasmid), 0.02 µM of forward primer and 0.06 µM of reverse primer of set 1, 0.01 µM of each forward and reverse primer of set 2, 0.03 µM of forward primer and 0.06 µM of reverse primer of set 3, 0.8 µM of the tag primer, 4 pmol of probe c.-78A>G, 7 pmol of probe c.52A>T, 4 pmol of probe c.125_128delTCTT, 7.5 pmol of probe c.216_217insA, 4 pmol probe of c.316-197C>T, 2 pmol of internal control probe, 3.0 mM MgCl_2_ in a PCR master mix (67 mM Tris-HCl, pH 8.0, 16.6 mM (NH_4_)_2_SO_4_, 6.7 µM EDTA, 0.085 mg/mL BSA, 2.0 U *TaKaRa Taq*, 300 µM dNTPs). The cycling was initiated by heating to 95°C for 5 min, continued by 10 cycles of touchdown PCR (95°C for 15 s, 65°C and touchdown -1°C each cycle for 2 min, 72°C for 20 s), and followed by another 45 cycles of 95°C for 15 s, 56°C for 20 s, and 72°C for 20 s. Fluorescence was recorded at the annealing steps during the second round of 45 cycles.

A blind study was implemented with 239 coded clinical samples (provided by Xiamen Maternity and Child Health Care Hospital) to valid the MCPC method. The exact genotypes of these samples were determined previously by reverse dot-blot analysis. β-thalassemia patient samples were also provided with clinical symptoms and parental genotypes in a coded format such that no personal information of the patients could be obtained.

## Supporting Information

Table S1 Specificity of MCPC-based real-time PCR in identification of 10 bacterial strains.
(DOC)

Table S2 Validation of MCPC assay in a blind test for β-globin mutations of 239 clinical samples.
(DOC)

Table S3 Sequences of primers and probes used for HPV typing.
(DOC)

Table S4 Sequences of primers and probes used for identification of 10 foodborne pathogens.
(DOC)

Table S5 Sequences of primers and probes used in the MCPC assay of β-globin alleles.
(DOC)
