# Supplementary material for: Multicolor Combinatorial Probe Coding for Real-Time PCR
Source: PLoS One. 2011 Jan 14;6(1):e16033. doi: 10.1371/journal.pone.0016033 (PMC3021529; doi:10.1371/journal.pone.0016033)
Supplement: Table S1 — Specificity of MCPC-based real-time PCR in identification of 10 bacterial strains. (DOC) [file pone.0016033.s001.doc]

**Table S1**. **Specificity of MCPC-based real-time PCR in identification of 10 bacterial strains**

| **Species** | **Number of**  **strains tested** | **qPCR result ^a^** | | | | | | | | | |
| --- | --- | --- | --- | --- | --- | --- | --- | --- | --- | --- | --- |
|  |  | Sal | O157 | Shi | Vp | Lm | Sa | Vc | Ye | Strep | Bc |
| *S. Typhi* | 15 | + | - | - | - | - | - | - | - | - | - |
| *E. coli* O157:H7 | 24 | - | + | - | - | - | - | - | - | - | - |
| *Shigella* spp. | 24 | - | - | + | - | - | - | - | - | - | - |
| *V. parahaemolyticus* | 10 | - | - | - | + | - | - | - | - | - | - |
| *L. monocytogenes* | 25 | - | - | - | - | + | - | - | - | - | - |
| *S. aureus* | 15 | - | - | - | - | - | + | - | - | - | - |
| *V. cholerae* | 24 | - | - | - | - | - | - | + | - | - | - |
| *Y. enterocolitica* | 2 | - | - | - | - | - | - | - | + | - | - |
| *S. pyogenes* | 1 | - | - | - | - | - | - | - | - | + | - |
| *B. cereus* | 3 | - | - | - | - | - | - | - | - | - | + |
| *R. equi* | 1 | - | - | - | - | - | - | - | - | - | - |
| *M. albican* | 1 | - | - | - | - | - | - | - | - | - | - |
| *V. mimicus* | 1 | - | - | - | - | - | - | - | - | - | - |
| *V. fluvialis* | 1 | - | - | - | - | - | - | - | - | - | - |
| *V. vulnificus* | 1 | - | - | - | - | - | - | - | - | - | - |
| *A. hydrophila* | 1 | - | - | - | - | - | - | - | - | - | - |
| *A. caviae* | 1 | - | - | - | - | - | - | - | - | - | - |
| *E. faecalis* | 1 | - | - | - | - | - | - | - | - | - | - |
| *Enteropathogenic E. coli* | 1 | - | - | - | - | - | - | - | - | - | - |
| *Enterohemorrhagic E. coli* | 1 | - | - | - | - | - | - | - | - | - | - |
| *B. subtilis* | 1 | - | - | - | - | - | - | - | - | - | - |
| *P. vulgaris* | 1 | - | - | - | - | - | - | - | - | - | - |
| *S. Faecalis* | 1 | - | - | - | - | - | - | - | - | - | - |
| *P. aeruginosa* | 1 | - | - | - | - | - | - | - | - | - | - |

^a^ +,positive; -, negative. Abbreviations for the bacteria: Sal, *Salmonella Typhi*; O157, *E. coli* O157:H7; Shi, *Shigella* spp; Vp, *V. parahaemolyticus*; Lm, *L. monocytogenes*; Sa, *S. aureus*; Vc, *V. cholerae*; Ye , *Y. enterocolitica*; Strep, *S. pyogenes*; Bc , *B. cereus*.
