# Supplementary material for: Multicolor Combinatorial Probe Coding for Real-Time PCR
Source: PLoS One. 2011 Jan 14;6(1):e16033. doi: 10.1371/journal.pone.0016033 (PMC3021529; doi:10.1371/journal.pone.0016033)
Supplement: Table S2 — Validation of MCPC assay in a blind test for β-globin mutations of 239 clinical samples. (DOC) [file pone.0016033.s002.doc]

**Table S2. Validation of MCPC assay in a blind test for β-globin mutations of 239 clinical samples**

| **Signature**  **number** | **Genotype** | **MCPC signature^a^** | | | | **Number of samples** |
| --- | --- | --- | --- | --- | --- | --- |
|  |  | **FAM** | **HEX** | **ROX** | **CY5** |  |
| wild-type and heterozygous carrier | | | | | | |
| 1 | Wild-type | 0 | 0 | 0 | 2 | 129 |
| 2 | c.316-197C>T | 1 | 0 | 0 | 2 | 44 |
| 3 | c.52A>T | 0 | 1 | 0 | 2 | 12 |
| 4 | c.-78A>G | 0 | 0 | 1 | 2 | 8 |
| 5 | c.216_217insA | 1 | 0 | 1 | 2 | 2 |
| 6 | c.125_128delTCTT | 0 | 1 | 1 | 2 | 32 |
| homozygous and compound heterozygous mutations | | | | | | |
| 7 | c.[316-197C>T]+[316-197C>T] | 2 | 0 | 0 | 2 | 1 |
| 11 | c.[125_128delTCTT]+[316-197C>T] | 1 | 1 | 1 | 2 | 2 |
| 13 | c.[-78A>G]+[52A>T] | 0 | 1 | 1 | 2 | 2 |
| 15 | c.[125_128delTCTT]+[52A>T] | 0 | 2 | 1 | 2 | 2 |
| 18 | c.[125_128delTCTT]+[-78A>G] | 0 | 1 | 2 | 2 | 2 |
| 20 | c.[125_128delTCTT]+[216_217insA] | 1 | 1 | 2 | 2 | 2 |
| 21 | c.[125_128delTCTT]+[125_128delTCTT] | 0 | 2 | 2 | 2 | 1 |
| Total | 239 | | | | | |
| ^a^ In the signature, “0” represents no signal, “1” represents signal from one haploid, “2” represents signal either from two haploids or from one haploid detected by two different probes but labeled with the same fluorophore. | | | | | | |
