# Supplementary material for: Multicolor Combinatorial Probe Coding for Real-Time PCR
Source: PLoS One. 2011 Jan 14;6(1):e16033. doi: 10.1371/journal.pone.0016033 (PMC3021529; doi:10.1371/journal.pone.0016033)
Supplement: Table S3 — Sequences of primers and probes used for HPV typing. (DOC) [file pone.0016033.s003.doc]

**Table S3**. **Sequences of primers and probes used for HPV typing**

| **Name** | **Sequence, 5' →3'** |
| --- | --- |
| **Primers**  GP5+  GP6+ | TTTGTTACTGTGGTAGATACTAC  GAAAAATAAACTGTAAATCATATTC |
| **Probes** |  |
| HPV6 | HEX-TCTTCCACATACACCA-PO_4_  /GTGTATGTGGAAGA-Dabycl |
| HPV11 | Cy5-CTGTGTCTAAATCTGCT-PO_4_  /CAGATTTAGACACAG-Dabycl |
| HPV16 | ROX-TGCTGCCATATCTACT-PO_4_  /TAGATATGGCAGCA-Dabycl |
| HPV18 | FAM-TCTCCTGTACCTGGG-PO_4_  /CAGGTACAGGAGA-Dabycl |
| HPV31 | FAM, ROX-CAATTGCAAACAGTGAT-PO_4_  /CACTGTTTGCAATTG-Dabycl |
| HPV33 | FAM, Cy5-TGACTTTATGCACACA-PO_4_  /TGTGCATAAAGTCA-Dabycl |
| HPV35 | FAM, HEX-TCTGTGTGTTCTGCT-PO_4_  /CAGAACACACAGA-Dabycl |
| HPV42 | ROX, Cy5-ACTGCAACATCTGGT-PO_4_  /CAGATGTTGCAGT-Dabycl |
| HPV52 | HEX, ROX-CTTTATGTGCTGAGGT-PO_4_  /CTCAGCACATAAAG-Dabycl |
| HPV58 | HEX, Cy5-CACTGAAGTAACTAAGGA-PO_4_  /CTTAGTTACTTCAGTG-Dabycl |
| HPV39 | FAM, ROX, Cy5-CCTCTATAGAGTCTTCCA-PO_4_  /GAAGACTCTATAGAGG-Dabycl |
| HPV43 | FAM, HEX, ROX-GACCCTACTGTGCC-PO_4_  /CACAGTAGGGTC-Dabycl |
| HPV44 | FAM, HEX, Cy5-CCACTACACAGTCCC-PO_4_  /GACTGTGTAGTGG-Dabycl |
| HPV45 | HEX, ROX, Cy5-CTAATTTAACATTATGTGCC-PO_4_  /CACATAATGTTAAATTAG-Dabycl |
| HPV40 | FAM, HEX, ROX, Cy5-ACACAGTCCCCCA-PO_4_  /GGGGACTGTGT-Dabycl |
