# Supplementary material for: Multicolor Combinatorial Probe Coding for Real-Time PCR
Source: PLoS One. 2011 Jan 14;6(1):e16033. doi: 10.1371/journal.pone.0016033 (PMC3021529; doi:10.1371/journal.pone.0016033)
Supplement: Table S4 — Sequences of primers and probes used for identification of 10 foodborne pathogens. (DOC) [file pone.0016033.s004.doc]

**Table S4**. **Sequences of primers and probes used for identification of 10 foodborne pathogens**

| **Bacterium strains** | **Target gene** | **Sequence, *^a^* 5' →3'** |
| --- | --- | --- |
| *Salmonella Typhi*  *E. coli O157:H7*  *Shigella*  *B.cereus*  *V. parahaemolyticus*  *L. monocytogenes*  *S.aureus*  *V. cholerae*  *S. pyogenes*  *Y. enterocolitica* | ssaR  rfbE  ipaH  ces  tdh  hly  nuc  ctxA  spy1258  ail  Tag | gaacctggcctgaagacataaa ^a^  agtaatccaatccgaaatgcct ^b^  ROX-ccggctaactgactcaccgtaaatgccgg-Dabycl ^c^  Tag-aggtgaaggtggaatggttgtc ^a^  Tag-gcttgttctaactgggctaatc ^b^  HEX-cggccaaggattagctgtacataggccg-Dabycl ^c^  Tag-tgaaggaaatgcgtttctatg ^a^  Tag-agggagaaccagtccgtaaa^b^  Cy5-cacggccgaagctatggtcagaagccgtg-Dabycl ^c^  Tag-aaactggatgccgttagaaca ^a^  Tag-aagccatcttgtcggttctg ^b^  FAM-cggcgttctcacttgtacttgatttcgccg-Dabycl ^c^  Tag-aaacatctgcttttgagcttcca ^a^  Tag-ctcgaacaacaaacaatatctcatcag ^b^  FAM, HEX-ccggggtgtcccttttcctgcccccgg-Dabycl ^c^  Tag-tgcaagtcctaagacgcca ^a^  Tag-cactgcatctccgtggtatactaa ^b^  ROX, Cy5-cgcgcttgtatatacttatcgatttcatccgcgcg-Dabycl ^c^  Tag-ggcaatacgcaaagaggtt ^a^  Tag-ccacttctatttacgccgttatc ^b^  FAM, Cy5-cgatgcagtctaagtagctcagcaaatgcatcg-Dabycl ^c^  Tag-tccggagcatagagcttgga ^a^  Tag-tcgatgatcttggagcattcc ^b^  HEX, ROX-ccgtggattcatcatgcaccgccacgg-Dabycl ^c^  Tag-ccgcactcgctactatttctta ^a^  Tag-attggtcacaatgtcttggaaac ^b^  FAM, ROX-ccgcagaaatccttgatgagttgcgg-Dabycl ^c^  Tag-ggttatgcgcaaagccatgt ^a^  Tag-ccctgatgagtataagcaaacga^b^  HEX, Cy5-ctccccgttatgaactcgatgataactggggag-Dabycl ^c^  gcaagccctcacgtagcgaa |

^a^ forward primer, ^b^ reverse primer, ^c^ probe
