# Supplementary material for: Multicolor Combinatorial Probe Coding for Real-Time PCR
Source: PLoS One. 2011 Jan 14;6(1):e16033. doi: 10.1371/journal.pone.0016033 (PMC3021529; doi:10.1371/journal.pone.0016033)
Supplement: Table S5 — Sequences of primers and probes used in the MCPC assay of β-globin alleles. (DOC) [file pone.0016033.s005.doc]

**Table S5. Sequences of primers and probes used in the MCPC assay of β-globin alleles**

| **Primers (5'→3')** | ^a^**Probes (5'→3')** | **Mutations** |
| --- | --- | --- |
| Tag-CCAATCTACTCCCAGGAGCA ^b^  Tag-CACCTTGATACCAACCTGCC ^c^  Tag-CCTTAGGCTGCTGGTGGTCT ^b^  Tag-TTTCCCACCCTTAGGCTGC ^c^  Tag-CATCATGCCTCTTTGCACCA ^b^  Tag-GCAATATGAAACCTCTTACATCAG ^c^  Tag: GCAAGCCCTCACGTAGCGAA | ROX-TGCCCTGACTTCTATGCCCAG-PO_4_  /TGGGCATAGAAGTCAGGGCA-Dabycl  HEX-CCTGTGGGGCTAGGTGAACGT-NH_2_  /CGTTCACCTAGCCCCACAGG-Dabcyl  ROX/HEX-AGGACTCAACCTCTGGGTCC-PO_4_  /GACCCAGAGGTTGAGTCCT-Dabcyl  ROX/ FAM-GTGCCTTTAAGTGATGGC-PO_4_  /CCATCACTTAAAGGCAC-Dabcyl  CY5-CTCCTGATGCTGTTATGGGCAAC**-**PO_4_  /GCCCATAACAGCATCAGGAG-DABCYL  FAM-TGGGTTAAGGTAATAGCAATA-NH_2_  /ATTGCTATTACCTTAACCCA-Dabcyl | c.-78A>G  c.52A>T  c.125_128delTCTT  c.216_217insA  Internal control  c.316-197C>T |

^a^ Underlined bases denotes LNA modifications, ^b^ forward primer, ^c^ reverse primer
